# Predicting feed intake using modelling based on feeding behaviour in finishing beef steers

**DOI:** 10.1016/j.animal.2021.100231

**Published:** 2021-07

**Authors:** C. Davison, J.M. Bowen, C. Michie, J.A. Rooke, N. Jonsson, I. Andonovic, C. Tachtatzis, M. Gilroy, C-A. Duthie

**Affiliations:** a Department Electronic and Electrical Engineering, University of Strathclyde, 204 George Street, Glasgow G1 1XW, UK; b Scotland’s Rural College, Beef and Sheep Research Centre, SRUC, West Mains Road, Edinburgh EH9 3JG, UK; c College of Medical, Veterinary and Life Sciences, University of Glasgow, Bearsden G61 1QH, UK; d Afimilk UK Ltd, Baltic Chambers, 50 Wellington Street, Glasgow G2 6HJ, UK

**Keywords:** Beef cattle, DM intake, Feed efficiency, Finishing steers, Machine learning

## Abstract

Current techniques for measuring feed intake in housed cattle are both expensive and time-consuming making them unsuitable for use on commercial farms. Estimates of individual animal intake are required for assessing production efficiency. The aim of this study was to predict individual animal intake using parameters that can be easily obtained on commercial farms including feeding behaviour, liveweight and age. In total, 80 steers were used, and each steer was allocated to one of two diets (40 per diet) which consisted of (g/kg; DM) forage to concentrate ratios of either 494:506 (**MIXED**) or 80:920 (**CONC**). Individual daily fresh weight intakes (**FWI**; kg/day) were recorded for each animal using 32 electronic feeders over a 56-day period, and individual DM intakes (**DMI**; kg/day) subsequently calculated. Individual feeding behaviour variables were calculated for each day of the measurement period from the electronic feeders and included: total number of visits to the feeder, total time spent at the feeder (**TOTFEEDTIME**), total time where feed was consumed (**TIMEWITHFEED**) and average length of time during each visit to the feeder. These feeding behaviour variables were chosen due to ease of obtaining from accelerometers. Four modelling techniques to predict individual animal intake were examined, based on (i) individual animal TOTFEEDTIME relative expressed as a proportion of the dietary group (**GRP**) and total GRP intake, (ii) multiple linear regression (**REG**) (iii) random forests (**RF**) and (iv) support vector regressor (**SVR**). Each model was used to predict CONC and MIXED diets separately, giving eight prediction models, (i) GRP_CONC, (ii) GRP_MIXED, (iii) REG_CONC, (iv) REG_MIXED, (v) RF_CONC, (vi) RF_MIXED, (vii) SVR_CONC and (viii) SVR_MIXED. Each model was tested on FWI and DMI. Model performance was assessed using repeated measures correlations (*R*^2^_RM) to capture the repeated nature of daily intakes compared with standard *R*^2^, RMSE and mean absolute error (**MAE**). REG, RF and SVR models predicted FWI with *R*^2^_RM = 0.1–0.36, RMSE = 1.51–2.96 kg and MAE = 1.19–2.49 kg, and DMI with *R*^2^_RM = 0.13–0.19, RMSE = 1.15–1.61 kg and MAE = 0.9–1.28 kg. The GRP models predicted FWI with *R*^2^_RM = 0.42–0.49, RMSE = 2.76–3.88 kg and MAE = 2.46–3.47 kg, and DMI with *R*^2^_RM = 0.32–0.44, RMSE = 0.32–0.44 kg, MAE = 1.55–2.22 kg. Whilst more simplistic GRP models showed higher *R*^2^_RM than regression and machine learning techniques, these models had larger errors, likely due to individual feeding patterns not being captured. Although regression and machine learning techniques produced lower errors associated with individual intakes, overall precision of prediction was too low for practical use.

## Implications

Improving feed efficiency is crucial for reducing the environmental impact and economic cost of beef production, whilst ensuring sustainable food production for a growing global population. Breeding for improved feed efficiency requires accurate measurement techniques for feed intake on an individual animal basis. These individual measurement techniques are currently not commercially accessible due to the cost and labour requirements of the systems. This study shows liveweight and feeding behaviour variables alone, which could be easily obtained from neck or ear mounted accelerometers (e.g. collars/ear tags), are not adequate proxies for estimating feed intake at an individual animal level.

## Introduction

Livestock production is under continued political pressure to reduce its greenhouse gas output, with enteric methane accounting for approximately 10–12% of global anthropogenic emissions (International Panel on Climate Change, 2014). The global human population is expected to exceed nine billion by 2050 with meat consumption projected to increase by more than 70% compared with 2010 levels (McLeod, 2011). Achieving this level of production, whilst reducing the environmental impact of ruminant livestock production, represents a considerable challenge. Feed represents the single largest variable cost of beef production (between 50 and 75%), and ruminant methane production represents a significant energy loss to the animal. Increasing the efficiency of converting feed into product (i.e. beef) will increase profitability, reduce the environmental impact and ultimately increase the sustainability of beef production systems.

In order to improve feed efficiency, it is important to accurately characterise the input (DM intake; **DMI**) and output (liveweight gain; **LWG**) on an individual animal basis. Current techniques for measuring individual animal feed intake are labour intensive and too expensive for commercial use. Although measuring individual animal feed intake in housed conditions is easier than outdoor scenarios, current systems are limited to testing stations or research environments and can service only 2–5 animals per individual unit (e.g. electronic feed intake recorders such as HokoFarm-Group (Insentec, Hokofarm Group, Marknesse, Netherlands) and GrowSafe (Airdrie, Alberta, Canada). Outdoor measurements at pasture (grazing) are restricted to indigestible markers, for example, the n-alkane technique (Mayes et al., 1986). This technique is labour intensive and prone to analytical errors, often resulting in poor estimations of intake (Laredo et al., 1991).

Results from feeding experiments conducted in different regions illustrate the large variation that exists in feed intake within groups of animals offered the same feed. For example, Fitzsimons et al. (2013) reported a difference of 15% in DMI in beef heifers, whilst Hyslop et al. (2014) found a 16% difference in DMI in finishing beef steers when comparing top and bottom terciles (i.e. most and least efficient animals). Furthermore, less efficient steers consumed £28 more feed (per head) over a 12-week finishing period compared to the most efficient tercile of animals (Hyslop et al., 2014). Alongside the large between animal variation in intake that exists, day to day intra-animal variation in intake in beef cattle also exists due to environmental factors (e.g. temperature; Koknaroglu et al., 2008).

Due to the large between animal variation in feed consumed, there is a need for proxy measures of DMI. Time spent eating, and other feeding behaviour variables, have previously been reported as being positively correlated to DMI in beef cattle (Nkrumah et al., 2007) and dairy cows (De Mol et al., 2016). These studies focused on assessing relationships (correlations) between feeding behaviour, feed intake and efficiency. However, to date, there have been no attempts to predict feed intake from feeding behaviour and readily available animal information in finishing (housed) beef cattle. Commercial systems already exist which are able to monitor feeding behaviour of individual animals, for example through animal mounted accelerometers. The aim of this study was to develop different analytical techniques to estimate individual feed intake using feeding behaviour, liveweight and age, measures readily available on farm with commercially available technologies. It is hypothesised that models which are trained on feed behaviours from individual animals will be able to learn non-linear relationships between these behaviours and resultant feed intake, and thus outperform models which rely on herd-level data. Preliminary results from this work have been previously presented as conference abstracts (Bowen et al., 2020).

## Material and methods

### Experimental design, diets and animals

The experiment was of a continuous design, comprising of two diets (concentrate- or silage-based) and one cross-bred breed type (purebred Limousin sire mated with Aberdeen Angus cross-bred dam). Two diets (fed as total mixed rations) were generated using a diet mixing wagon and consisted of (g/kg DM) forage to concentrate ratios of either 494:508 (**MIXED**) or 80:920 (**CONC**). The diets were selected to represent contrasting commercially applicable diets.

The DM contents of individual diet components were determined on duplicate samples twice weekly. Bulked feed samples were analysed for DM, ash, crude protein, neutral detergent fibre, acid hydrolysed ether extract, starch, and neutral cellulose and gammanase digestibility (Ministry of Agriculture Fisheries and Food, 1992), and metabolisable energy was estimated (Thomas, 2004). The ingredient and chemical compositions of the experimental diets are given in Table 1.

**Table 1:** Ingredient composition and chemical composition of cattle fed CONC (concentrate based total mixed ration) or MIXED (mixed forage/concentrate based total mixed ration) diets.

Item	CONC	MIXED
Components (g/kg DM)		
Grass Silage	–	169
Wholecrop Barley Silage	–	325
Barley Straw	80	–
Barley Grain	650	272
Maize Distillers Dark Grains	239	202
Molasses (cane)	21	22
Minerals	10	10
Composition (g/kg DM)		
DM (g/kg)	836	432
CP	122	143
NDF	331	341
Starch	133	95
AHEE	43	48
Ash	41	66
Metabolisable Energy (MJ/kg DM)	12.3	12.3
NCGD (%)	80	79

Abbreviations: AHEE = acid hydrolysed ether extract; NCGD = neutral cellulose gammanase digestibility.

In total, 80 steers were used (40 per diet) and each diet was allocated to two pens (four pens in total; 20 steers per pen). Pens were balanced for sire, farm of origin and liveweight and were balanced across diets at the start of the experiment. Fresh water was provided *ad libitum* using a water trough, and diets were offered at approximately 1.05 times average daily intake to all steers using 32 electronic feeders (HOKO, Insentec, Marknesse, The Netherlands; eight electronic feeders per pen) to ensure *ad libitum* access to feed. All steers were bedded on wood fibre and sawdust to ensure that consumption of bedding did not contribute to nutrient intake and influence feeding behaviours. All steers were fed the MIXED diet before being adapted to diets. Steers allocated the CONC diet were gradually adapted to the full concentrate inclusion over a 4-week period. Forage to concentrate ratios were increased at weekly intervals such that ratios of 38:62, 25:75, 13:87 and 8:92 were offered during adaptation. During this period, steers were trained to use the electronic feed intake recording equipment.

### Feeding behaviour, growth and performance measures

Individual daily fresh weight intakes (**FWI**, kg/day) were recorded for each animal using the electronic feeding equipment and DMI (kg/day) subsequently calculated. After adaptation to the experimental diets, feeding behaviour was monitored for all steers over a 56-day test period. One animal from the CONC dietary treatment was removed due to ill-health unrelated to the study. Animals were maintained under controlled conditions, where group sizes within the pen remained constant. Feeding behaviour variables were calculated for each day of the 56-day trial period, generating 3 693 days’ worth of observations. Feeding behaviour variables calculated include total number of visits to the feeder each day (**TOTVISIT**), total time spent at the feeder calculated as the sum of time of entry to time of departure for each visit daily (**TOTFEEDTIME**), total time where intake was consumed calculated as sum of time spent at the feeder where intake was recorded (**TIMEWITHFEED**) and average length of time during each visit to the feeder (**AVVISITLENGTH**). Feeding behaviours were recorded on a per visit basis and summarised daily, and daily summaries of variables were used in subsequent analysis. Steers were weighed weekly on a calibrated weigh scale, and the liveweight on the nearest weigh day was used for daily liveweights during the 56-day trial period (**LIVEWEIGHT**). Feeding rate was not included in these models as prior knowledge of feed intake is required.

### Statistical analysis

Diet effects on FWI and DMI and feeding behaviour were assessed using the Wilcoxon rank test (R Studio, V 3.4.3) due to the non-normal distribution of the data (normality assessed using qq plots in R Studio). Due to differences observed in feeding behaviour between dietary treatments (Table 2), all subsequent analysis and modelling were performed separately for CONC and MIXED diets. The relationship between feeding behaviour variables (TOTVISIT, TOTFEEDTIME, TIMEWITHFEED and AVVISITLENGTH) and feed intake was assessed using repeated measure correlation (Python Pingouin, V0.2.9) as described by (Bakdash and Marusich, 2017), to account for both variability within the individual and variability across the herd.

**Table 2:** Means and SD for feeding behaviour variables (by day) between cattle offered CONC (concentrate based total mixed ration) and MIXED (mixed forage/concentrate based total mixed ration) diets.

	CONC		MIXED		
Item	Mean	SD	Mean	SD	*P*-value
Fresh Weight Intake (kg/day)	13.49	2.22	23.24	3.35	<0.001
DM Intake (kg/day)	11.85	1.95	10.5	1.55	<0.001
TOTVISIT (number/day)	32.28	11.86	44.96	20.04	<0.001
TOTFEEDTIME (min/day)	96.05	20.44	136.30	22.30	<0.001
TIMEWITHFEED (min/day)	94.38	19.46	134.94	21.94	<0.001
AVVISITLENGTH (min/day)	3.31	1.20	3.62	1.62	<0.001

Abbreviations: TOTVISIT = total number of visits to the feeder, TOTFEEDTIME = total time spent at the feeder, TIMEWITHFEED = time at the feeder where intake was consumed, AVVISITLENGTH = average length of time during each visit to the feeder.

### Prediction models to estimate intake

Four techniques for predicting and estimating intakes (FWI and DMI) were created based on (i) an animal’s proportion of TOTFEEDTIME relative to the TOTFEEDTIME and total fresh weight intake of the group (**GRP**) (**TOTFEEDTIMEGRP**), (ii) traditional multiple linear regression based on feeding behaviours, age and LIVEWEIGHT (**REG**), and two machine learning techniques (iii) random forests (**RFs**) and (iv) support vector regressor (**SVR**). Machine learning (RF and SVR) and REG models were used to develop models based on training datasets. Both *R*^2^ and repeated measure correlation (*R*^2^_RM; Bakdash and Marusich, 2017) were used to assess model performance due to the repeated predictions within animals. RMSE and mean absolute errors (**MAE**s) were calculated for each model. MAE is more suitable to represent the average deviation, while RMSE indicates the influence of outliers on the overall error.

$$\mathrm{MAE}=\frac{\sum_{i=1}^{n}\left|y_{i}-\hat{y_{i}}\right|}{n}$$

Errors (RMSE and MAE) were also calculated to assess errors associated with *R*^2^_RM, in which errors were calculated for each individual and averaged to provide repeated measures RMSE (**RMSE_RM**) and MAE (**MAE_RM**).

*GRP_CONC and GRP_MIXED.* In these prediction models, group FWI was estimated using the sum of all individual feed intakes *f*_group_, TOTFEEDTIME of an individual animal and TOTFEEDTIMEGRP of the group. This is defined in the equation below, where PI is the predicted FWI (kg), *f*_group_ is the sum of all individual animal intakes each day (kg), *t*_ind_ is TOTFEEDTIME of an individual animal (min) and *t*_group_ is TOTFEEDTIMEGRP of the group (min). This model was repeated to predict DMI.

$$\mathrm{PI}=f_{group}\frac{t_{ind}}{t_{group}}$$

*REG_CONC and REG_MIXED.* In these prediction models, data were split into a training dataset containing 75% of the data and test dataset containing the remaining 25%. Data were split based on animals and both datasets were balanced to account for pen differences. Multiple linear regression models were created using the lm() function in R Studio with FWI and DMI as response variables. Variables in the model included TOTVISIT, TOTFEEDTIME, TIMEWITHFEED, AVVISITLENGTH, AGE, LIVEWEIGHT and average daily temperature (DAILYTEMP) taken from meteorological data at a local weather station. Each model was tested using the corresponding dataset.

*RF_CONC and RF_MIXED.* These prediction models were based upon random forest, a supervised machine learning technique (Breiman, 2001). As with other supervised learning techniques, data are split into a training dataset to build the model and test dataset to assess model performance. Random forests are built from a series of decision trees, each built from a random subset of the samples and variables from the training dataset (Breiman, 2001). When each decision tree is built using the training dataset, about one-third of samples are left out, this is known as the out-of-bag data. After each tree is built, the out-of-bag data are run down the decision tree to assess model performance and to calculate variable importance (described further below). The final result of the model is calculated by averaging the outcome of all decision trees (in the case of regression). Individually, each decision tree is poor at accurate predictions, however, combining multiple trees allows for vast improvements in prediction.

Due to differences in feeding behaviour observed between dietary treatments, random forest models were built for CONC (**RF_CONC**) and MIXED (**RF_MIXED**) diets separately. For each diet, data were split to create a training dataset (75% of the original dataset) and test dataset (25%) of the data based on individual animals, e.g. 30 animals were included in the training dataset and 10 in the test dataset, with training and test datasets balanced for pen differences RF_CONC and RF_MIXED models were created using the randomForest package in R studio (V 3.4.3), with DMI and FWI as response variables. Variables included in RF_CONC and RF_MIXED models include TOTVISIT, TOTFEEDTIME, TIMEWITHFEED, AVVISITLENGTH, AGE, LIVEWEIGHT and DAILYTEMP. Models were tuned by adjusting the number of trees (ntree) used, and the number of variables used at each split (mtry). To determine the optimum ntree in each random forest model, 3 500 trees were grown and the ntree with the lowest mean squared error value (from OOB samples) was selected, allowing for mean square errors to first be stabilised. The mtry was set as the square root of the number of variables. The final ntree parameters for predicting DMI were 3 487 and 3 499 for RF_CONC and RF_MIXED models, respectively. The final ntree for predicting FWI was 685 and 2 383 for RF_CONC and RF_MIXED, respectively. The mtry was set as three for RF_CONC and RF_MIXED models for both FWI and DMI. The variable importance was assessed using mean decrease in accuracy (%IncMSE), which is calculated by permutating an individual variable whilst untouching the remaining variables (i.e. removing one of the variables in the out-of-bag samples and replacing with a random one) and comparing it against the unpermuted variable. This is repeated for each variable used in the random forest models and plotted in order of importance – a higher %IncMSE value represents a higher variable importance within the model. Each model was tested using the corresponding test dataset.

*SVR_CONC and SVR_MIXED.* Support vector models using non-linear kernels are able to capture complex relationships between datapoints during training and are able to achieve good performance when training data are of limited volume. The SVR (Adwad and Khanna, 2015) is a reformulation of the SVM algorithm for application to regression problems. Given training samples x, the aim is to determine a function such that predicted value $\hat{x}$ falls with ε of the true value $f\left(x\right)$; where ε represents an error tolerance. Any predictions that lie outside the tolerance contribute towards the loss function. To avoid model overfitting, Tikhonov regularization is utilised. By modifying the kernel used by the SVR algorithm, it is possible to model both linear and non-linear relationships. In this research, the radial-bias function kernel was utilised to allow modelling of non-linear relationships between variables. Due to the difference in feeding behaviours between the CONC and MIXED diets, models were trained separately using the SupportVectorRegressor module (Scikit-Learn (Python), v0.20.3). For the SVR model, 75% of the animals (30 steers) were used for training dataset and 25% (10 steers) in the test dataset, balancing for differences between pens. Average performance was evaluated through 5-fold Monte Carlo cross-validation. The target variables were DMI and FWI, with TOTVISIT, TOTFEEDTIME, AVVISITLENGTH, AGE and LIVEWEIGHT as input features. Due to the usage of a non-linear kernel, it was not possible to retrieve feature importance of either SVR model.

## Results

### Animal information and diet effects on feeding behaviour

Steers had a mean (SD) age of 476 (41) and 475 (42) days at the start of the trial period for CONC and MIXED diets, respectively. Initial starting liveweight was 536 (61) kg for animals on CONC and 529 (64) for MIXED diets. The LWG for the 56-day trial period was 1.60 (0.22) and 1.26 (0.20) for CONC and MIXED diets, respectively.

Steers offered the MIXED diet had higher FWI (*P* < 0.001), higher TOTVISITS (*P* < 0.001), longer TOTFEEDTIME (*P* < 0.001), longer TIMEWITHFEED (*P* < 0.001) and longer AVVISITLENGTH (*P* < 0.001) and lower DMI (*P* < 0.001) compared to the CONC diet (Table 2).

Repeated measure correlations were used to assess correlations between feeding behaviours and feed intake to more accurately capture the repeated nature of daily intakes. Correlations between TOTFEEDTIME and daily FWI for both the CONC and MIXED diets were respectively 0.609 (*P* < 0.001) and 0.604 (*P* < 0.001). The correlation was lower between AVVISITLENGTH and daily FWI, *r* = 0.478 (*P* < 0.001) for the CONC and *r* = 0.274 (*P* < 0.001) for the MIXED diet. The TOTVISIT only showed a weak correlation with the MIXED diet, at *r* = 0.165 (*P* < 0.001), and no correlation with the CONC diet (*r* = −0.044; *P* < 0.001). The calculated correlation coefficients were identical when using DMI as Pearson correlation is invariant to a change in scale.

### Accuracy of prediction models and variable importance

When considering FWI, GRP_CONC and GRP_MIXED models showed highest *R*^2^_RM (0.42 and 0.49, respectively). Whilst these models showed highest accuracy, errors were largest for these models (RMSE_RM = 2.76 and 3.88 kg and MAE_RM = 2.46 and 3.47 kg). Regression based models showed a slight reduction in accuracy for REG_CONC (*R*^2^_RM = 0.35) and REG_MIXED (*R*^2^_RM = 0.36); however, errors were decreased (Table 3). Machine learning based models showed lower *R*^2^_RM (SVR_CONC = 0.24; SVR_MIXED = 0.20; RF_CONC = 0.18; RF_MIXED = 0.10); however, these models had lower errors (RMSE and MAE; Table 3). Similarly, when considering DMI, GRP_CONC and GRP_MIXED showed highest *R*^2^_RM values but also had highest errors compared to machine learning techniques (Table 3).

**Table 3:** Performance measures of proportion of group feeding behaviour (GRP), regression based model (REG), random forest (RF) and support vector regressor (SVR) models for predicting DMI and fresh weight intake (FWI). All prediction models were created separately within cattle offered CONC (concentrate based total mixed ration) and MIXED (mixed forage/concentrate based total mixed ration) diets.

	*R*^2^	*P*	RMSE	MAE	*R*^2^_RM	*P*	RMSE	MAE
FWI								
GRP_CONC	0.02	<0.001	3.13	2.46	0.42	<0.001	2.76	2.46
GRP_MIXED	0.11	<0.001	4.32	3.48	0.49	<0.001	3.88	3.47
REG_CONC	0.44	<0.001	1.56	1.23	0.35	<0.001	1.53	1.22
REG_MIXED	0.36	<0.001	2.76	2.25	0.36	<0.001	2.65	2.45
RF_CONC	0.55	<0.001	1.84	1.64	0.18	<0.001	1.40	1.19
RF_MIXED	0.46	<0.001	3.11	2.49	0.10	<0.001	2.96	2.49
SVR_CONC	0.54	<0.001	1.56	1.18	0.24	<0.001	1.51	1.19
SVR_MIXED	0.48	<0.001	2.44	1.94	0.20	<0.001	2.38	1.94
DMI								
GRP_CONC	0.01	<0.001	2.83	2.22	0.32	<0.001	2.54	2.23
GRP_MIXED	0.12	<0.001	1.95	1.55	0.44	<0.001	1.76	1.55
REG_CONC	0.44	<0.001	1.37	1.07	0.34	<0.001	1.34	1.06
REG_MIXED	0.35	<0.001	1.28	1.05	0.30	<0.001	1.23	1.05
RF_CONC	0.55	<0.001	1.61	1.28	0.18	<0.001	1.59	1.27
RF_MIXED	0.44	<0.001	1.45	1.19	0.13	<0.001	1.40	1.19
SVR_CONC	0.51	<0.001	1.43	1.01	0.19	<0.001	1.38	1.06
SVR_MIXED	0.44	<0.001	1.15	0.90	0.19	<0.001	1.12	0.91

Abbreviations: *R*^2^_RM = *R*^2^ using repeated measure correlations, MAE = mean absolute error.

### Variable importance

Variable importance was calculated for RF models. LIVEWEIGHT (4.0; 5.2 %IncMSE), TIMEWITHFEED (1.6; 2.0 %IncMSE) and AGE (1.0; 1.4 %IncMSE) were the three most important variables for predicting FWI and DMI respectively within the CONC diet. The most important variables within the MIXED diet were LIVEWEIGHT (2.2; 10.1 %IncMSE), AGE (0.8; 4.1 %IncMSE) and TOTVISIT (0.7; 3.3 %IncMSE) for predicting FWI and DMI, respectively.

## Discussion

The objective of this study was to develop and assess various prediction models based on feeding behaviour and animal size (e.g. liveweight) as a proxy for estimating individual animal feed intake. Accurate recordings of feed intake are essential to measure, with the ultimate goal of improving, feed efficiency in cattle. The development of such models has the potential to overcome current issues associated with the cost and accessibility of measurement of individual feed intakes in a commercial setting. In this paper, models that incorporate individual feeding behaviour were contrasted against models that work with herd-level aggregates. Models using individual feeding behaviour (REG, RF and SVR) were shown to have lower prediction error (RMSE_RM and MAE_RM) compared to the simple group model operating on herd-level behaviours (GRP). These results suggest that models which characterise the individual may prove beneficial, however, more work is needed to reduce the error and thus enable use in a commercial setting.

### Feeding behaviour and correlations with intake

Time spent at the feeder (TOTFEEDTIME) was comparable with other studies (Nkrumah et al., 2007, Haskell et al., 2019, Parsons et al., 2020), where beef steers were fed high concentrate diets through electronic feeders. The TOTFEEDTIME was also comparable with steers fed a mixed diet (Haskell et al., 2019). Previous studies have reported positive correlations between feeding behaviours (e.g. TOTFEEDTIME) and feed intake in both dairy and beef animals, suggesting models which can learn individual behaviours may deliver improved prediction. Although lower than results reported in this study, Nkrumah et al. (2007) reported positive correlations (*r* = 0.27; *P* < 0.001) between daily feeding time and DMI in steers fed a high concentrate diet. Similarly, positive correlations (*r* = 0.13; *P* < 0.05) between time at the feeder and DMI have been reported in beef steers fed a high corn (Montanholi et al., 2010) and high concentrate (*r* = 0.42; *P* < 0.05; Parsons et al., 2020) diets, although a negative correlation (*r* = −0.16; *P* < 0.05) with number of visits to the feeder was also noted (Montanholi et al., 2010). Higher correlations between feeding time and FWI (*r* = 0.53–0.95) have been noted in dairy cows fed a total mixed ration (De Mol et al., 2016, Pahl et al., 2016). Feeding is regulated by negative feedback signals from gut-fill to avoid over stretching of the gut wall and metabolic signals based on availability of nutrients (Forbes and Gregorini, 2015). Cattle fed higher forage diets (MIXED) are likely influenced greater by gut-fill regulation due to the bulkiness of the food explaining the increase in TOTVISIT and TOTFEEDTIME observed in animals offered the MIXED diet compared to CONC. Other factors including hierarchy and group dynamics around feed bin space may also play a role.

### Models to predict individual animal intake

Several recent studies have attempted to predict feed intake of individual animals based on physiological measurements in both dairy and beef cattle. Data from the RumiWatch System (RumiWatch, Liestal, Switzerland) and IGER behaviour recorders (Ultra Sound Advice, London, UK), used to quantify grazing behaviour, were combined with physiological measurements (including liveweight, body measurements, linear type scoring and thermal imaging) to estimate intake in grazing lactating beef cows with moderate success. Results (*R*^2^ = 0.59; Williams et al., 2019) were similar to values reported in this study. However, it must be noted that *R*^2^_RM values, although showing lower accuracy than *R*^2^, provide a better idea of model performance due to the repeated measure nature of the data. A machine learning approach using boosted regression trees achieved moderate correlations (*r* = 0.73) between actual and predicted intakes in dairy cows (Kamphuis et al., 2017), and the *R*^2^ was not reported. A second machine learning approach utilised artificial neural networks to predict daily FWI with RMSE of 1.72 (Van der Waaij et al., 2016), similar to errors reported from machine learning techniques for FWI in this study. These studies have focused on using physiological measurements to predict intake. To date, no study has attempted to predict intake from feeding behaviour variables, which can be easily obtained from neck, or ear mounted accelerometers, and basic animal information.

The present study has quantified the error in feed intake estimate as a number of kg/day. This is a meaningful parameter for a farm operative wishing to understand the production performance of an individual. Hence, it is more meaningful to consider the MAE_RM and the RMSE_RM, with MAE_RM ranging from 0.91 to 1.27 kg and 1.19 to 2.49 kg on DMI and FWI, respectively, and RMSE_RM ranging from 1.12 to 1.59 kg and 1.40 to 2.96 kg on DMI and FWI, respectively. The feed intake estimated from percentage of time spent feeding in relation to other animals in the group (GRP) gives the highest RMSE_RM and MAE_RM for both diets whether expressed as DMI (RMSE_RM 1.76–2.54 kg, MAE_RM 1.55–2.23 kg) or FWI (RMSE_RM 2.76–3.88, MAE_RM 2.46–3.47). The higher error in the group model is because the individual animal feeding preferences (including feeding rates) are not captured when estimating feed as a proportion of time in relation to the group. In addition, prediction techniques using REG, RF and SVM were based on data split into training and test datasets, which allows the model to be created and tuned prior to prediction. These datasets were also split based on individual animals which allowed variation between animals across the entire trial period to be accounted for. Both the RF and the SVR can weight contributions from a range of measurements that influence feed intake and consequently produce an estimate that is closer to the individual performance.

### Use of technology

Measurements of feed intake at an individual animal level are desirable because they can be used to calculate the production efficiency of an individual and hence enable farm operatives to make informed operational decisions, e.g. when is the optimum time to replace an animal. Automated measurement of feed intake using electronic feeder systems is possible in research environments (Chizzotti et al., 2015) but such systems are not practical for commercial farms. Collar and/or ear tag monitoring devices which contain accelerometers can provide measurements of time spent feeding. These are derivatives of automated oestrus detection systems which are commonplace in dairy farming (Afimilk, 2015; Heat Detection and Health Monitoring - National Milk Records, 2018). As market competition for oestrus detection aids has increased, manufacturers have sought to differentiate their products by expanding their services. Consequently, feeding and rumination durations were targeted because they can be related to animal welfare. Feeding and rumination behaviour can be detected by processing accelerometer signals (Martiskainen et al., 2009, Kok et al., 2015, Smith et al., 2016, Michie et al., 2017). FScore classifications of 0.8 for (Smith et al., 2016) are reported for both collars and ear tag devices, indicating a strong balance between precision and recall when detecting these behaviours. Similar performances are reported for commercially available systems (Borchers et al., 2016). Feeding and rumination behaviour provides useful information and is used to alert to welfare issues such as lameness (Thorup et al., 2016) or other welfare disorders (Mottram, 2016, Stangaferro et al., 2016). However, no commercial systems currently exist that use collars or ear tags to estimate individual animal feed intake (DMI or FWI). While researchers have reported the correlation between time spent feeding and feed intake (De Mol et al., 2016), no study has quantified the precision of estimating feed intake using measurements of time spent feeding alone. Here, prediction models were created from feeding behaviours which are readily accessible from animal mounted accelerometers (e.g. collar or ear tag technology). The TOTFEEDTIME can be measured using collars or ear tags. From this, the time that an individual or the herd spends feeding can be calculated. In addition to TOTFEEDTIME, the TOTVISIT along with the AVVISITLENGTH can give an insight into individual feeding preferences. External parameters such as the animal age, liveweight and the average daily temperature were used as inputs.

The above analysis has quantified the range of errors that may take place when attempting to estimate feed intake using measurements of feeding time. The approach that has been taken in the present work was to calculate the scale of the errors under ideal conditions, i.e. where exact measurements of feeding time are available. In a production setting, inaccuracies in practical tools such as collars and ear tags will further influence the robustness of the above methods. This was not considered in the present analysis which aims to provide a baseline characterisation to inform the discussion as to whether such tools are valuable in this context. In conclusion, with a mean error of approximately 10% of daily intake, this study has shown that feeding behaviour and liveweight alone are not sufficient to accurately predict feed intake on an individual animal level.

## Ethics statement

This study was conducted at SRUC’s Beef and Sheep Research Centre situated six miles south of Edinburgh UK. The experiment was approved by the Animal Experiment Committee of SRUC and was conducted in accordance with the requirements of the UK Animals (Scientific Procedures) Act 1986.

## Data and model availability statement

None of the data were deposited in an official repository. The data that support the findings of this study are available upon reasonable request from the corresponding author.

## Author ORCIDs

**Christopher Davison:** https://orcid.org/0000-0002-9450-1791.
**Jenna Bowen:** https://orcid.org/0000-0001-7398-8353.
**Craig Michie:** https://orcid.org/0000-0001-5132-4572.
**John Rooke:** https://orcid.org/0000-0002-9780-9804.
**Nicholas Jonsson:** https://orcid.org/0000-0003-3245-9783.
**Ivan Andonovic:** https://orcid.org/0000-0001-9093-5245.
**Christos Tachtatzis:** https://orcid.org/0000-0001-9150-6805.
**Carol-Anne Duthie:** https://orcid.org/0000-0003-3020-9078.

## Author Contributions

**Christopher Davison:** Methodology, Formal Analysis, Writing – Original Draft. **Jenna Bowen:** Methodology, Formal Analysis, Writing – Original Draft. **Craig Michie:** Funding Acquisition, Conceptualization, Project Administration, Writing – Review and Editing. **John Rooke:** Data Curation, Methodology, Writing – Review and Editing. **Nicholas Jonsson:** Writing – Review and Editing. **Ivan Andonovic:** Funding Acquisition, Conceptualization, Project Administration, Writing – Review and Editing. **Christos Tachtatzis:** Writing – Review and Editing. **Michael Gilroy:** Funding Acquisition, Conceptualization, Writing – Review and Editing. **Carol-Anne Duthie:** Funding Acquisition, Conceptualization, Data Curation, Methodology, Project Administration, Writing – Review and Editing.

## Declaration of interest

None to declare.

## References

[b0005] Adwad, M., Khanna, R., 2015. Support vector regression. In Efficient Learning Machines (ed M Awad and R Khanna). Apress, Berkeley, CA, USA, pp. 67–80. https://doi.org/10.1007/978-1-4302-5990-9_4.

[b0010] Bakdash J.Z., Marusich L.R. (2017). Repeated measures correlation. Frontiers in Psychology.

[b0015] Borchers M.R., Chang Y.M., Tsai I.C., Wadsworth B.A., Bewley J.M. (2016). A validation of technologies monitoring dairy cow feeding, ruminating, and lying behaviors. Journal of Dairy Science.

[b0020] Bowen, J.M., Davison, C., Michie, C., Duthie, C.-A., 2020. Using machine learning techniques to estimating feed intake from feeding behaviour and liveweight in finishing beef steers. In Proceedings of the British Society of Animal Science (BSAS), 30 March to 1 April 2020, East Midlands Conference Centre, UK, pp. 104.

[b9000] Breiman L. (2001). Random Forests. Machine Learning.

[b0025] Chizzotti M.L., Machado F.S., Valente E.E.L., Pereira L.G.R., Campos M.M., Tomich T.R., Coelho S.G., Ribas M.N. (2015). Technical note: Validation of a system for monitoring individual feeding behavior and individual feed intake in dairy cattle. Journal of Dairy Science.

[b0030] Fitzsimons C., Kenny D.A., Deighton M.H., Fahey A.G., McGee M. (2013). Methane emissions, body composition, and rumen fermentation traits of beef heifers differing in residual feed intake. Journal of Animal Science.

[b0035] Forbes J.M., Gregorini P. (2015). The catastrophe of meal eating. Animal Production Science.

[b0040] Haskell M.J., Rooke J.A., Roehe R., Turner S.P., Hyslop J.J., Waterhouse A., Duthie C.A. (2019). Relationships between feeding behaviour, activity, dominance and feed efficiency in finishing beef steers. Applied Animal Behaviour Science.

[b0045] Hyslop J.J., Fuller R., Taylor U., Thirlwell D., Wareing S. (2014). Feed intake, animal performance and net feed efficiency (NFE) in finishing Stabiliser steers. Proceedings of the British Society of Animal Science (BSAS), 29 and 30 April 2014, Nottingham, UK.

[b0050] International Panel on Climate Change, 2014. Climate Change 2014: Synthesis Report. Contribution of Working Groups I, II and III to the Fifth Assessment Report of the Intergovernmental Panel on Climate Change. Intergovernmental Panel on Climate Change, Geneva, Switzerland.

[b0055] Kamphuis C, Van Riel JW, Veerkamp RF and De Mol RM 2017. Traditional mixed linear modelling versus modern machine learning to estimate cow individual feed intake. Proceedings of European Conference on Precision Livestock Farming, 12 to 14 September 2017, Nantes, France, pp. 366–376.

[b0060] Kok A., van Knegsel A.T.M., van Middelaar C.E., Hogeveen H., Kemp B., de Boer I.J.M. (2015). Technical note: Validation of sensor-recorded lying bouts in lactating dairy cows using a 2-sensor approach. Journal of Dairy Science.

[b0065] Koknaroglu H., Otles Z., Mader T., Hoffman M.P. (2008). Environmental factors affecting feed inake of steers in different housing systems in the summer. International Journal of Biometeorology.

[b0070] Laredo M.A., Simpson G.D., Minson D.J., Orpin C.G. (1991). The potential for using n- alkanes in tropical forages as a marker for the determination of dry matter by grazing ruminants. The Journal of Agricultural Science.

[b0075] Martiskainen P., Jarvinen M., Skon J.P., Tiirikainen J., Kolehmainen M., Mononen J., Järvinen M., Skön J.-P., Tiirikainen J., Kolehmainen M. (2009). Cow behaviour pattern recognition using a three-dimensional accelerometer and support vector machines. Applied Animal Behaviour Science.

[b0080] Mayes R.W., Lamb C.S., Colgrove P.M. (1986). The use of dosed and herbage n-alkanes as markers for the determination of herbage intake. Journal of Agricultural Science, Cambridge.

[b0085] McLeod A. (2011). World Livestock 2011 - Livestock in food security.

[b0090] Michie C., Andonovic I., Tachtatzis C., Davison C., Konka J., Uttamchandani D. (2017). Wireless MEMS sensors for precision farming. Wireless MEMS Networks and Applications.

[b0095] Ministry of Agriculture Fisheries and Food, 1992. Ministry of Agriculture Fisheries and Food. Analysis of Agricultural Materials, 2nd edition. Her Majesty’s Stationary Office, London, UK.

[b0100] De Mol R.M., Goselink R.M.A., Van Riel J.W., Knijn H.M., Van Knegsel A.T.M., Kamphuis C., Steeneveld W. (2016). The relation between eating time and feed intake of dairy cows. Precision Dairy Farming 2016.

[b0105] Montanholi Y.R., Swanson K.C., Palme R., Schenkel F.S., McBride B.W., Lu D., Miller S.P. (2010). Assessing feed efficiency in beef steers through feeding behavior, infrared thermography and glucocorticoids. Animal.

[b0110] Mottram T. (2016). Animal board invited review: Precision livestock farming for dairy cows with a focus on oestrus detection. Animal.

[b0115] Nkrumah J.D., Crews D.H., Basarab J.A., Price M.A., Okine E.K., Wang Z., Li C., Moore S.S. (2007). Genetic and phenotypic relationships of feeding behavior and temperament with performance, feed efficiency, ultrasound, and carcass merit of beef cattle. Journal of Animal Science.

[b0120] Pahl C., Hartung E., Grothmann A., Mahlkow-Nerge K., Haeussermann A. (2016). Suitability of feeding and chewing time for estimation of feed intake in dairy cows. Animal.

[b0125] Parsons I.L., Johnson J.R., Kayser W.C., Tedeschi L.O., Carstens G.E. (2020). Characterization of feeding behavior traits in steers with divergent residual feed intake consuming a high-concentrate diet. Journal of Animal Science.

[b0130] Smith D., Rahman A., Bishop-Hurley G.J., Hills J., Shahriar S., Henry D., Rawnsley R. (2016). Behavior classification of cows fitted with motion collars: Decomposing multi-class classification into a set of binary problems. Computers and Electronics in Agriculture.

[b0135] Stangaferro M.L., Wijma R., Caixeta L.S., Al-Abri M.A., Giordano J.O. (2016). Use of rumination and activity monitoring for the identification of dairy cows with health disorders: Part III. Metritis. Journal of Dairy Science.

[b0140] Thomas, C., 2004. Feed into Milk. A new applied feeding system for dairy cows. Nottingham University Press, Nottingham, UK.

[b0145] Thorup V.M., Nielsen B.L., Robert P.-E., Giger-Reverdin S., Konka J., Michie C., Friggens N.C. (2016). Lameness affects cow feeding but not rumination behavior as characterized from sensor data. Frontiers in Veterinary Science.

[b0150] Van der Waaij B.D., Feldbrugge R.L., Veerkamp R.F., Kamphuis C., Steeneveld W. (2016). Cow feed intake prediction with machine learning. Precision Dairy Farming 2016.

[b0155] Williams M., Prendiville R., O’Sullivan K., McCabe S., Kennedy E., Liddane M., Buckley F. (2019). Developing and validating a model to predict the dry matter intake of grazing lactating beef cows. Animal.

